# Prevalence of multidrug-resistant and extended-spectrum β–lactamase-producing *Escherichia coli* from chicken farms in Egypt

**DOI:** 10.14202/vetworld.2023.1001-1007

**Published:** 2023-05-13

**Authors:** Gamal A. Salem, El-Sayed A. Abdelaziz, Mohammed A. Kamel, Nasreddin R. Rhouma, Reem I. Ali

**Affiliations:** 1 Department of Pharmacology, Faculty of Veterinary Medicine, Zagazig University, Zagazig 44511, Egypt; 2 Department of Microbiology, Faculty of Science, Misurata University, Misurata 2478, Libya

**Keywords:** antimicrobial resistance, beta-lactamase, chicken farms, *Escherichia coli*

## Abstract

**Background and Aim:**

Extended-spectrum beta-lactamase (ESBL)-producing *Escherichia coli* strains exhibit antibiotic resistance and are known to infect humans worldwide. This study assessed the phenotypic and genotypic prevalence of ESBL-resistant *E. coli* isolates recovered from the respiratory tracts of chickens in El-Sharkia Governorate, Egypt.

**Materials and Methods:**

We obtained 250 lung samples (one lung/bird) from 50 chicken farms (5 chickens/farm) to isolate, identify, and serotype *E. coli*. Antimicrobial resistance susceptibility was determined using the disk diffusion method, while the ESBL phenotype was identified using double disk synergy. We detected the β-lactamase genes, *bla*TEM, and *bla*SHV, using a polymerase chain reaction.

**Results:**

The results showed that 140/250 (56%) were infected with *E. coli*. All the serogroups of isolated *E. coli* exhibited high multi-antimicrobial resistance index values (>0.2), and 65.7% were confirmed to have ESBL. Among the isolates with the ESBL phenotypes, 55 (60%) and 32 (35%) contained the *bla*TEM and *bla*SHV genes, respectively.

**Conclusion:**

The widespread distribution of multidrug-resistant and ESBL-producing *E. coli* among poultry farms is a significant human health hazard. These results will help the Egyptian authorities to implement a national one-health approach to combat the antimicrobial resistance problem.

## Introduction

Meat production from chicken, one of the most frequently farmed animals, accounts for over 90 billion tons annually worldwide [1]. The antibiotics used to treat bacterial infections in humans and food-producing animals can also be used as growth promoters to improve the productivity of poultry. However, the treatment efficacy of antibiotics is hindered by the rapid spread of antibiotic-resistant (ABR) pathogens. In the US, over 2.8 million ABR bacterial infections have been reported, with a mortality rate of approximately 35,000 [2]. Reports have shown that using antibiotics at sub-therapeutic concentrations in poultry feed increases their production [3]. As growth promoters, antibiotics exert several beneficial effects, such as combating subclinical diseases, reducing illness and mortality rates, enhancing growth rates, minimizing feed costs, and increasing the feed conversion ratio [4, 5]. Certain antibiotic families are used as growth promoters in poultry and livestock, including penicillins, aminoglycosides, and tetracyclines. These are also used to treat bacterial infections in humans [6]. Excessive and inconsistent use of antibiotics in animals and humans has led to the emergence of muti-drug-resistant bacterial strains that can spread antibiotic resistance globally [7].

Avian pathogenic *Escherichia coli* (APEC) causing systemic or local infections outside the gut are known as extraintestinal pathogenic *E. coli* (ExPEC). Colibacillosis due to ExPEC affects broiler chicken aged 4–6 weeks and is characterized by sub-acute fibrinous aracialities, peritonitis, pericarditis, salpingitis, or septicemia [8, 9]. Colibacillosis negatively impacts poultry production by increasing costs due to treatment and prophylaxis, mortality rates, and rejection of diseased carcasses at slaughterhouses [9]. Avian pathogenic *E. coli* exhibits various resistance patterns against antibiotics approved for poultry, including penicillin, sulfonamides, chloramphenicol, tetracyclines, fluoroquinolones, and aminoglycosides [10]. Extended-spectrum beta-lactamase (ESBL) producers are resistant to penicillin and oxyimino-cephalosporins, such as cefotaxime, ceftazidime, and monobactams. However, they cannot degrade cephamycins and are inactivated by clavulanic acid [11]. Several drugs permitted for veterinary medicine have been implicated in the development of ESBL-producing Gram-negative Enterobacteriaceae [12, 13], contributing to the emergence of ESBL genes among APEC strains [14, 15]. Antibiotic-resistant commensal *E. coli* strains can distribute the ABR genes to other pathogenic bacteria, spreading resistant genes from poultry to humans [16]. In addition to chicken farms, ESBL-producing *E. coli* strains have also been found in other farm animals and meat products [17, 18]. The most ubiquitous ESBL genes, TEM and SHV [19], have been found in food-producing animals, indicating that the food chain might be a possible transmission route from animals to humans [15, 20].

Hence, monitoring the ABR patterns of human pathogens and pathogenic commensal bacteria in animals is crucial. In this study, we aimed to examine the prevalence of multidrug-resistant and ESBL-producing *E. coli* and perform genotypic characterization of the ESBL-related beta-lactamase (*bla*) genes, including *bla*TEM and *bla*SHV in poultry farms in the El-Sharkia Governorate, Egypt.

## Materials and Methods

### Ethical approval

This study was conducted according to the guidelines of the Institutional Animal Care and Use Committee of Zagazig University, Egypt (Approval no. ZU-IACUC/12/F/5/2019).

### Study period and location

The study was conducted from December 2019 to April 2021 at the Faculty of Veterinary Medicine, Zagazig University, and the Reference Laboratory for Quality Control on Poultry Production (RLQP), Dokki, Egypt.

### Sample collection, bacterial isolation, and identification

We collected 250 samples (lungs and trachea) from birds exhibiting clinical signs and post-mortem lesions of colibacillosis from 50 poultry farms, including commercial, intensive, low-scale (5000–10,000 birds), and rural farms with poor biosecurity levels and no official veterinary supervision from different regions in El-Sharkia Governorate, Egypt, between December 2019 and April 2021. The antibiotics used in these poultry farms either as growth promoters and prophylactics or for treatment included bacitracin, colistin, tetracycline, amoxicillin, cefotaxime, ampicillin, doxycycline, lincomycin, spectinomycin, fluorophenol, and ceftiofur Na.

After aseptically collecting the samples in sterile bags, they were immediately transported to the Reference Laboratory for Quality Control on Poultry Production (RLQP), Dokki, for bacteriological examination. To identify the *E. coli* strains, 25 g of the samples were homogenized in 250 mL of buffered peptone water and incubated at 37°C for 24 h for pre-enrichment [21]. Then, the strains were isolated and identified using a technique recommended by Swayne [22].

### Serological identification of *E. coli*

According to Edwards and Ewing [23], the isolated strains were serotyped at the Animal Health Research Institute, Dokki, Giza, using diagnostic polyvalent and monovalent *E. coli* antisera (Denka Seiken Co. Ltd, Japan).

### Antimicrobial susceptibility

The antimicrobial susceptibility of all *E. coli* isolates was tested using agar diffusion against 13 antibiotics (Oxoid, Hampshire, UK) according to Clinical and Laboratory Standards Institute guidelines and was clinically categorized with breakpoints [24]. Multi-resistant isolates, that is, resistant to three or more antimicrobial categories, were selected for further examination [25].

### Phenotypic screening of ESBL

We cultured suspensions containing 0.5 McFarland units of the confirmed multi-resistant strains on Mueller-Hinton agar plates (Oxoid) with cefotaxime and ceftazidime disks. The inhibition zone surrounding the disk/tablet with cephalosporin alone was compared to the zone around the disk/tablet containing cephalosporin and clavulanic acid. A size difference of ≥5 mm between the zones formed the disks with clavulanic acid and those without represents positive results [26].

### Bacterial DNA extraction

The isolates were streaked on nutrient agar and incubated for 14–16 h at 37°C. Then, 5 mL liquid culture media was inoculated with a single colony and incubated overnight at 37°C. Genomic DNA was extracted at the RLQP using the G-spin™ Total DNA Extraction Kit (INTRON Biotechnology, Korea) according to the manufacturer’s recommendations.

### Detection of ABR genes

The polymerase chain reaction (PCR) oligonucleotide primers were obtained from Metabion (Germany) and Biobasic (Canada) (Table-1) [27]. The 25 μL-PCR reaction mix consisted of 12.5 μL of Emerald Amp Max PCR Master Mix (Takara, Japan), 6 μL of template DNA, 1 μL of 20 pmol of each primer, and 4.5 μL of water. The PCR reactions were conducted using the Applied Biosystems 2720 thermal cycler under the following conditions: Initial denaturation for 3 min at 95°C; 30 cycles of 95°C for 30 s; annealing at a specific temperature for 1 min; extension at 72°C for 30 s; and final extension at 72°C for 5 min. To analyze the products, 15 μl of each product was loaded into each lane of a 1.5% agarose gel (Applichem, Darmstadt, Germany). Electrophoresis was performed in 1× Tris Boric acid EDTA buffer at a 5 V/cm gradient. The fragment sizes were determined using a 100 bp DNA ladder (Fermentas, Waltham, Massachusetts, USA). The gel photographs were obtained and analyzed using a gel documentation system (Alpha Innotech, Biometra, Germany).

**Table-1:** Oligonucleotide primers sequences sources.

Gene	Primer sequence (5’- 3’)	Length of amplified product	Reference
*bla*TEM	ATCAGCAATAAACCAGC	516 bp	[27]
	CCCCGAAGAACGTTTTC		
*Bla*SHV	AGGATTGACTGCCTTTTTG	392 bp	
	ATTTGCTGATTTCGCTCG		

## Results

### Prevalence of *E. coli* among the examined poultry samples

Of the 250 chickens examined for *E. coli* infections from 50 broiler poultry farms, 140 specimens (56%) were positive for *E. coli*, while 110 were negative (44%) (Table-2).

**Table-2:** Incidence of *E. coli* isolated from chickens.

Bacterial spp.	Number of farms	Number of samples	Results			

			+ve	%	−ve	%
*Escherichia coli*	50	250	140	56	110	44

The percentage was calculated according to the total number of farms. *E. coli*=*Escherichia coli*

### Serotyping of *E. coli* isolates

The *E. coli* isolates were serotyped using eight specific polyvalent and 43 monovalent group O somatic antisera. The results are shown in Table-3.

**Table-3:** Serotyping of *E. coli* strains isolated from poultry samples.

Monovalent *E. coli* serogroups	No.	%	Polyvalent *E. coli* serogroups
O86a	25	17.8	1
O55	10	7.1	2
O166	5	3.5	2
O111	25	17.8	1
O125	50	35.7	2
O127	10	7.1	1
O157	15	10.7	3

### Sensitivity of *E. coli* serotypes to different antibiotics

The antibiotic resistance of the 140 *E. coli* strains was tested against 14 antibiotics using the disk diffusion method. As shown in Table-4, *E. coli* O groups were 100% resistant to streptomycin, cephalexin, oxytetracycline, and doxycycline, followed by ampicillin (94.6%) and sulphamethoxazole-trimethoprim, cefotaxime, and ceftazidime (92.9%). Furthermore, 24 out of 140 *E. coli* strains were resistant to apramycin, and 115 strains (82.1%) were resistant to norfloxacin, ciprofloxacin, and colistin sulfate.

**Table-4:** The percentage of sensitivity and resistance of 140 *E. coli* serovars isolated from chickens against 13 antimicrobial disks.

Antimicrobial disk	Resistant		Intermediate		Sensitive	
		
	No.	%	No.	%	No.	%
Ampicillin	135	96.4	5	3.5	0	0
Chloramphenicol	80	100	0	0	0	0
Norfloxacin	115	82.1	5	3.6	20	14.3
Ciprofloxacin	115	82.1	10	7.1	15	10.8
Oxytetracycline	140	100	0	0	0	0
Sulphamethazole-trimethoprim	130	92.9	5	3.6	5	3.6
Colistin sulphate	115	82.1	0	0	25	17.9
Apramycin	120	85.7	20	14.3	0	0
Streptomycin	140	100	0	0	0	0
Cephalexin	140	100	0	0	0	0
Cefotaxime	130	92.9	10	7.1	0	0
Ceftazidime	130	92.9	0	0	10	7.1
Doxycycline	140	100	0	0	0	0

### Multidrug resistance and phenotypic ESBL production in the isolated *E. coli* strains

Tables-5 and 6 show the multiple antimicrobial resistance (MAR) index and ESBL production in the *E. coli* strains isolated from the chickens.

**Table-5:** Multiple antimicrobial resistances index analysis of *E. coli* isolates.

*Escherichia coli* isolates	No.	No. of antibiotics to which the isolate was resistant (a)	MAR index (a/b)	Phenotypic ESBL production
O111	2	12	0.92	−ve
O157	15	11	0.85	+ve
O125	7	12	0.92	+ve
O86a	4	13	1	−ve
O125	10	13	1	+ve
O125	6	11	0.8	+ve
O111	2	11	0.85	−ve
O55	4	13	1	−ve
O127	7	7	0.57	+ve
O166	5	12	0.9	+ve
O125	6	13	1	−ve
O111	9	12	0.92	−ve
O86a	6	13	1	−ve
O125	4	13	1	+ve
O55	6	12	0.92	+ve
O86a	5	11	0.92	−ve
O125	5	9	0.71	−ve
O125	3	11	0.85	+ve
O127	3	13	1	+ve
O111	6	13	1	−ve
O125	4	12	0.92	+ve
O86a	7	11	0.85	+ve
O125	2	13	1	−ve
O127	7	13	1	−ve
O86a	3	10	0.78	+ve
O125	1	11	0.85	+ve
O125	2	11	0.85	+ve
O111	9	11	0.85	+ve

No. of antimicrobials to which the isolates were subjected=13 (b). *E. coli*=*Escherichia coli*, MAR=Multiple antimicrobial resistance, ESBL=Extended-spectrum beta-lactamase

**Table-6:** The phenotypic ESBL production in *E. coli* isolates.

Bacterial spp.	ESBL	Non ESBL	Total
*Escherichia coli*	92 (65.7%)	48 (34.2%)	140

ESBL=Extended-spectrum beta-lactamase

### Detection of the *bla*SHV gene

**Figure-1:**
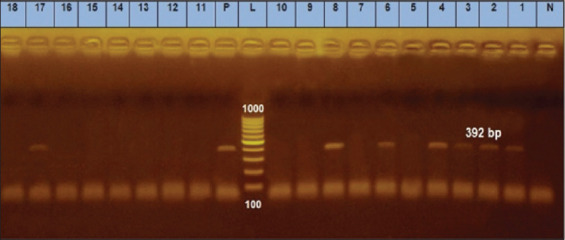
Agarose gel electrophoresis of polymerase chain reaction for detection of *bla*SHV gene in *Escherichia coli* isolates showing amplification of 392 bp in examined samples. L (Ladder): DNA ladder (100−1000 bp); Lanes 1–4, 6, 8, 17: positive samples. Pos=Positive control, Neg=Negative control.

The *bla*SHV gene imparts resistance against β-lactam antibiotics. Of the 92 ESBL-producing strains, 32 isolates (−35%) exhibited positive amplification of a 516 bp fragment using the primer specific to the *bla*SHV gene. The positive control also showed this 516 bp fragment, whereas the negative control showed no amplification (Figure-1).

### Detection of the *bla*TEM gene

**Figure-2:**
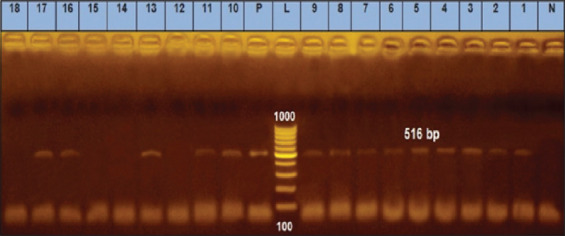
Agarose gel electrophoresis of polymerase chain reaction for detection of *bla*TEM gene in *Escherichia coli* isolates showing amplification of 516 bp in examined samples. L (Ladder): DNA ladder (100−1000 bp); Lanes 12, 14–15, 18: negative samples; Pos=Positive control; Neg=Negative control.

*Escherichia coli* strains containing the *bla*TEM gene are resistant to β-lactam antibiotics. We found that 60% of the *E. coli* isolates (55/92) with the ESBL phenotype contained this gene, as shown by the amplification of the 516 bp fragment using the primer specific for the *bla*TEM gene. The positive control also showed this 516 bp fragment, whereas the negative control did not (Figure-2).

## Discussion

Our study investigated the prevalence of multidrug-resistant pathogenic or commensal *E. coli* isolated from the respiratory tracts of infected chickens with chronic respiratory disease signs. We also detected the genes causing ABR against β-lactam antibiotics, which can be transferred to zoonotic or commensal bacteria and, subsequently, to humans, causing potential infections. Colibacillosis in chickens might be a primary or secondary infection, which induces diverse localized or systemic infections caused by *E. coli* [28, 29].

In this study, we found that *E. coli* was found in 56% of the chickens, possibly due to intensive rearing of birds in poorly aerated houses or because of *Mycoplasma galliseptecum* infection of the flock, which causes or aggravates colisepticaemia. Environmental stresses and respiratory viral infections may also predispose the chickens to this disease [30]. Similar results were shown by El-Tawab *et al*. [31], who reported a higher incidence of *E. coli* in chickens during winter (60.9%) than in summer (41%). These results agreed with that of Ruzauskas *et al*. [32], who reported a 41.7% prevalence of *E. coli* in raw chicken livers. Furthermore, similar results were obtained by Sarba *et al*. [33], who isolated *E. coli* from 40.4% of samples from colisepticaemia chickens.

Here, 140 of the 250 *E. coli* isolates obtained from chickens were categorized into seven O serogroups. The most dominant serotype was *E. coli* O 125, occurring in 35.7% of isolates (50/140), which is consistent with the previous studies of Ozaki *et al*. [34], who reported that O125 was the most prevalent (61.3%) serogroup associated with colibacillosis in poultry [33].

Due to the significant losses caused by colibacillosis in poultry production globally, different antibiotic groups are used to combat this infection, including β-lactams, aminoglycosides, fluoroquinolones, sulfonamides, and tetracyclines, which has led to the emergence of multi-resistant *E. coli* strains [35]. The antibiogram performed for different *E. coli* serotypes against 13 antibiotics revealed that approximately 100% of the *E coli* isolates were multi-resistant as they were resistant to at least three antibiotics, displaying a so-called MAR pattern (against ≥3 antimicrobials). *E. coli* isolates showed high resistance against 13 antibiotics. A previous study examining broiler chickens in Egypt detected a high phenotypic resistance rate of *E. coli* to penicillin, streptomycin, trimethoprim/sulphamethoxazole, and tetracycline [31, 36]. Other studies also showed the prevalence of multi-resistant *E. coli* among poultry farms in Tunisia [16] and Jordan [37].

Our results showed that 65.7% of the *E. coli* isolates were phenotypically ESBL-positive, consistent with Abdallah *et al*. [18], who found that 65.09% of *E. coli* isolates (69/106) were ESBL producers. However, a recent survey showed that 46.7% of the chicken in poultry farms (56/120) in Egypt were phenotypically positive for ESBL [15]. In Sweden, ESBL-producing *E. coli* was found in the guts of approximately 34% of broilers [38]. In Malaysia, 48.8% of the *E. coli* isolates obtained from retail chicken meat shops were ESBL-positive [39].

The *bla*TEM and *bla*SHV genes were prevalent in 50 (60%) and 30 (35%) ESBL-producing *E. coli*, respectively. Similarly, another study detected TEM and SHV genes in 57.55% [18] and 23.58% of the *E. coli* isolates, respectively, in Egypt. Further, reports have also shown a high incidence of *bla*TEM among ESBL-producing *E. coli* (94.73% [31] and 88.2% [27]). Contrastingly, relatively lower rates of *bla*TEM (9.1%) and *bla*SHV (1.8%) genes were detected by Ibrahim *et al*. [37] and Huijbers *et al*. [40], respectively.

Studies have found the *bla*TEM gene in 94.73% of isolated *E. coli* strains (18/19) [31], which agrees with the results of Colom *et al*. [27], who detected this gene in 88.2% of amoxicillin-clavulanate resistant *E. coli* isolates (45/51). However, these results contradicted with the study by Overdevest *et al*. [41], who obtained a lower percentage (−14%). The *bla*SHV and *bla*TEM genes were found in 1.8% and 72.9% of APEC isolates in Jordan, respectively [37]. This varies from the results of Huijbers *et al*. [42], who found that the incidence of *bla*SHV (17%) was higher than *bla*TEM (9.1%) among the ESBL-producing *E. coli* strains isolated from infected broilers and people employed in these farms [31].

ESBLs produced from Gram-negative bacteria, especially enterobacteriaceae, are encoded by plasmids and can cause resistance against all three generations of cephalosporins [43]. As cephalosporins are used to treat several Gram-negative bacterial infections in humans, the spread of ESBL-producing bacteria poses a significant public health threat to humans [44].

As antibiotic resistance is a global health issue [45], countries need to urgently adopt a national one approach to combat antimicrobial resistance, such as strengthening and implementing regulations for antibiotics use in veterinary and public health fields, focused surveillance to curb the spread of antimicrobial resistance in animals and humans, updating the guidelines of antimicrobial treatments, continuing education for prescribers on antimicrobial use, and monitoring drug abuse by animal owners and unskilled people for treating animals [46–48].

## Conclusion

This study examined the different *E. coli* serotypes isolated from chicken farms in the El-Sharkia Governorate, Egypt. We found that 100% of the strains were multidrug-resistant, of which 65.7% were ESBL producers. We also detected high rates of *bla*TEM and *bla*SHV genes among these isolates. These results suggest that these resistance genes can be potentially transferred to humans, causing a significant public health threat. We recommend that the Egyptian authorities plan and implement a national one-health approach to combat ABR, including widening the surveillance of antimicrobial resistance, enforcing the available regulations, and monitoring the production, storage, sale, and usage of veterinary drugs.

## Authors’ Contributions

GAS: Conceptualized the study, wrote the manuscript, and curated and analyzed the data. EAA and MAK: Supervised the study, validated the results, conceptualized the work, and reviewed and edited the manuscript. RIA: Conceived the work, performed all experiments, and contributed to writing of the manuscript. NRR: Analyzed the data and drafted and revised the manuscript. All authors have read, reviewed, and approved the final manuscript.
